# The prevalence of chronic ankle instability in basketball athletes: a cross-sectional study

**DOI:** 10.1186/s13102-022-00418-0

**Published:** 2022-02-18

**Authors:** Chiao-I Lin, Frank Mayer, Pia-Maria Wippert

**Affiliations:** 1 Medical Sociology and Psychobiology, Department of Physical Activity and Health, University of Potsdam, Am Neuen Palais 10, 14469 Potsdam, Germany; 2 University Outpatient Clinic, Centre of Sports Medicine, University of Potsdam, Am Neuen Palais 10, 14469 Potsdam, Germany; 3 Faculty of Health Sciences Brandenburg [Joint Faculty of the University of Potsdam, the Brandenburg Medical School Theodor Fontane and the Brandenburg University of Technology Cottbus – Senftenberg], Potsdam, Germany

**Keywords:** Functional ankle instability, Perceived ankle instability, Ankle sprain, Ankle injury, Survey, Basketball

## Abstract

**Background:**

Ankle sprain is the most common injury in basketball. Chronic ankle instability develops from an acute ankle sprain may cause negative effects on quality of life, ankle functionality or on increasing risk for recurrent ankle sprains and post-traumatic osteoarthritis. To facilitate a preventative strategy of chronic ankle instability (CAI) in the basketball population, gathering epidemiological data is essential. However, the epidemiological data of CAI in basketball is limited. Therefore, this study aims to investigate the prevalence of CAI in basketball athletes and to determine whether gender, competitive level, and basketball playing position influence this prevalence.

**Methods:**

In a cross-sectional study, in total 391 Taiwanese basketball athletes from universities and sports clubs participated. Besides non-standardized questions about demographics and their history of ankle sprains, participants further filled out the standard Cumberland Ankle Instability Tool applied to determine the presence of ankle instability. Questionnaires from 255 collegiate and 133 semi-professional basketball athletes (male = 243, female = 145, 22.3 ± 3.8 years, 23.3 ± 2.2 kg/m^2^) were analyzed. Differences in prevalence between gender, competitive level and playing position were determined using the Chi-square test.

**Results:**

In the surveyed cohort, 26% had unilateral CAI while 50% of them had bilateral CAI. Women had a higher prevalence than men in the whole surveyed cohort (X^2^(1) = 0.515, p = 0.003). This gender disparity also showed from sub-analyses, that the collegiate female athletes had a higher prevalence than collegiate men athletes (X^2^(1) = 0.203, p = 0.001). Prevalence showed no difference between competitive levels (p > 0.05) and among playing positions (p > 0.05).

**Conclusions:**

CAI is highly prevalent in the basketball population. Gender affects the prevalence of CAI. Regardless of the competitive level and playing position the prevalence of CAI is similar. The characteristic of basketball contributes to the high prevalence. Prevention of CAI should be a focus in basketball. When applying the CAI prevention measures, gender should be taken into consideration.

**Supplementary Information:**

The online version contains supplementary material available at 10.1186/s13102-022-00418-0.

## Background

Ankle sprain is one of the most common injuries among active individuals [1]. Residual symptoms after an acute ankle sprain such as pain, swelling, giving way or weakness are also prevalent [2]. In a follow-up 2.4 years after an acute ankle sprain, 74% of patients had residual symptoms [2]. The residual symptoms vary among the patients [3, 4]. The terms of this pathology are also diverse (e.g. chronic ankle instability (CAI), chronic ankle sprain or recurrent lateral ankle instability) [3, 5]. To standardize CAI for research purposes, the International Ankle Consortium characterized CAI as a pathology occurring among individuals who have a history of significant ankle sprains, experience “giving way,” and/or recurrent sprain, and/or “feelings of instability in the injured ankle” [3].

Based on the model from Van Mechelen, the first step of sports injuries prevention is to identify the severity and the incidence [6]. The severity of CAI is well documented [4]. CAI induces different short- to long-term negative consequences. For example, declined physical activity in long-term, decreased quality of life, recurring ankle sprain, early degenerative joint tissue changes and can potentially increase the load on the anterior cruciate ligament [4, 7]. In addition, recurrent ankle sprains induced by CAI cause financial burden and time loss [4]. To manage ankle sprains, direct costs range from 292 to 2268 USD per person and indirect costs are between 1482 and 4343 USD [8]. An ankle sprain results in 21–30 days of lost time for professional football athletes [9]. These negative consequences make the individual prone to further injuries, illness and affect the athletes’ time available for practices and games [4, 10]. To develop an injury prevention strategy for CAI and its negative consequences, injury surveillance is required [11].

Regarding the incidence of CAI, the epidemiology of ankle sprains is well documented. Yet the prevalence of CAI in basketball is not. Ankle sprain is one of the most common injuries in basketball due to repetitive jumping, cuttings, rapid stops and directional changes [12–14]. Incidences of ankle sprain among professional basketball athletes (National Basketball Association, the Women’s National Basketball Association and an elite Spanish basketball club) were between 1.3 and 4.3 per 1000 athlete exposures [15–17]; and among US collegiate athletes were 1.0–2.1 per 1000 athlete exposures [12, 18–20]. Lateral ankle sprain is the most common injury (16.2%) among US collegiate athletes [13].

Referring to the prevalence of CAI, 19–22% of all injuries in the basketball athlete population are recurrent ankle sprains [12, 18, 21]. In basketball, 60% of the participants experienced recurrent ankle sprain, 28% perceived ankle instability with a history of ankle sprain and 30% suffered from persistent symptoms after an ankle sprain [22]. In addition, 30% (17/57) of collegiate basketball athletes in the US and 4–64% (1/24, 8/22 and 14/22) in Japan had CAI [23–25]. Previous studies showed a wide range of the prevalence of CAI among basketball athletes (4% to 64%) and the sample sizes were small (N = 22–57) [23–25]. In addition, previous studies excluded athletes with a history of fractures and injuries in the lower extremities even though these athletes may also have CAI so the prevalence of CAI may be underestimated [26]. A survey for basketball athletes should be conducted and athletes with various other ankle issues should also be included to form an accurate picture of the prevalence of CAI.

Gender [15, 18, 21, 27], level of competition [19, 20], and position on court [18, 27] have been considered as factors that impact the ankle sprain rate in basketball. Women are considered prone to sports injuries due to physiological differences, e.g. joint laxity and menstrual cycles [28, 29]. At a higher competitive level, athletes have a higher level of compact body composition [30], intensity [19], athletic exposure [31] and rate of previous ankle sprains [32]. These factors may cause a higher rate of ankle injury compared to the athletes at a lower level [31]. Basketball athletes in different positions do different tasks [33]. Guards with good aerobic and anaerobic capacity perform high-intensive tasks [34]. Forwards run a lot during competition [33]. Centers, the tallest, heaviest and strongest among all basketball positions, carry out rebounding and have a lot of body contact with other opponents during boxouts [34]. The physiological profiles are distinct among play positions [35] and different characteristics may be associated with different injury rates [18]. However, the evidence of injury rate between genders [15, 18, 21], competitive levels [19, 20] and position [18, 27] are inconsistent. Once gender, level of competition and played position are correlated with the prevalence of CAI, the second, third and fourth steps of the model from Van Mechelen (establishing underlying mechanisms, developing and evaluating injury prevention programs) can be investigated and specialized [6, 36].

Therefore, the purposes of this study were to investigate the prevalence of CAI in elite basketball athletes at different levels (semi-professional and college) and to investigate if the prevalence of CAI is influenced by different genders, competitive levels and positions.

## Methods

### Study design and procedure

This study presents the cross-sectional data of CAI prevalence during the pre-season of the Super Basketball League and the games of the University Basketball Association in Taiwan. The investigator contacted team staff from all semi-professional teams and college teams in Taiwan. Participants filled out a printed questionnaire inquiring demographics, history of ankle sprain/giving way/recurring ankle sprain and the Taiwan-Chinese version of the Cumberland Ankle Instability Tool Questionnaire (CAIT-TW) after a routine practice [37]. The investigator distributed printed questionnaires and checked the questionnaires when returned it. Study was conducted in October of 2018. All participants read and signed the informed consent document before participating in the study. This study was performed in accordance with the Declaration of Helsinki and was approved by the ethical committee at the University of Potsdam (Number: 25/2018).

### Participants

391 elite Taiwanese basketball athletes from 11 semi-professional and 17 college teams were recruited for this study. 134 athletes (99 men and 35 women) from all the Super Basketball League in Taiwan (semi-professional level) (Additional File 1). 257 athletes (148 men and 109 women) from the University Basketball Association in Taiwan (teams ranked within the top ten in 2017) (Additional File 1). A convenient sampling was applied to select the college teams. Most of the included college teams are from the north of Taiwan. Inclusion criteria were: (1) participants were basketball team members and (2) a minimum age of 18 years. Exclusion criteria were (1) acute injuries in the lower extremities and (2) incomplete questionnaires. Athletes were also excluded if they could not participate in daily practice sessions due to injuries.

### Instruments

There were three sections in the questionnaire: (1) demographics, (2) history of significant ankle sprains/giving way/recurrent ankle sprain, and (3) the CAIT-TW [37].

1. The demographic section included age, gender, height, weight, training hours per week, training experiences, competitive level and playing position.
2. The questions stated:
  Have you sprained your ankle significantly? Which ankle? A significant ankle sprain was associated with inflammatory symptoms, interrupting at least 1 full day of planned physical activity, resulting in some initial deficits of function and disability [3].
  Have you experienced giving way? Giving way refers to regular occurrences of uncontrolled and unpredictable episodes of excessive inversion of the rear foot (usually experienced during initial contact during walking or running), which do not result in an acute lateral ankle sprain and it has happened at least twice in the past six months [3].
  Do you have recurrent ankle sprains? (two or more sprains to the same ankle) [3].
3. The CAIT-TW consisting of nine items was applied to determine the presence of perceived ankle instability [37]. CAIT-TW was culturally adapted from the original English Cumberland Ankle Instability Tool Questionnaire (CAIT) and evaluated the psychometric properties in an athletic population. The CAIT-TW showed excellent test–retest reliability (ICC_2.1_ = 0.91, p < 0.001), good internal consistency (Cronbach’s α: 0.87), moderate to strong construct validity (CAIT-TW versus Taiwan-Chinese version of Lower Extremity Functional Score: Rho = 0.39, p < 0.001 and strong (CAIT-TW versus Numeric Rating Scale: Rho = 0.76, p < 0.001), and a cutoff score of 21.5 (Youden index: 0.73, sensitivity: 0.87, specificity 0.85) [37].

### Data analysis

The International Ankle Consortium suggested the inclusion criteria of CAI are individuals who had a history of significant ankle sprain and either (1) experience of giving way twice or more within the past six months, (2) recurrent ankle sprain or (3) perceived ankle instability (the score of CAIT-TW is lower than 22) [3]. To reduce the recall bias on the experience of giving way and recurrent ankle sprain, athletes were considered to have CAI if they have a history of significant ankle sprain(s) and the presence of perceived ankle instability evaluated using CAIT-TW.

All data analysis was performed using IBM SPSS 25.0 (Chicago, Illinois, USA). Descriptive statistics were performed to display the demographic data and prevalence of CAI in the population of basketball athletes (first study purpose). Differences in demographics between participants with CAI and without CAI were examined applying the Mann–Whitney U test or independent T-test depending on the distribution of the data. The Chi-square test was applied to determine the difference in the presence of CAI between genders, two competitive levels and positions on court (second study purpose). The level of significance was set at p-value ≤ 0.05.

## Results

391 basketball athletes filled out the questionnaire in total, whereby three questionnaires were incomplete. Finally, 388 valid questionnaires were available for analysis (men = 243, women = 145, college = 255, semi-professional = 133). Participants’ demographics and the score of CAIT-TW are shown in Additional File 1 (22.3 ± 3.8 years, 179.9 ± 10.9 cm, 76.1 ± 13.7 kg). In the surveyed cohort, 97% of the participants experienced ankle sprain, 26% of them had unilateral CAI while 50% had bilateral CAI and 24% of them were without CAI (Additional File 1). There were no demographical differences between participants with CAI and without CAI (Table 1). The demographic data was separated by genders at different competitive levels and presented in Additional files 2, 3, 4.

**Table 1:** Demographical differences between participants with and without chronic ankle instability

	CAI (n = 297)	Without CAI (n = 91)	Difference between groups
	M ± SD	M ± SD	
Age (year)	22.4 ± 3.8	21.9 ± 3.8	p = 0.31
Height (cm)	179.5 ± 11.1	181.5 ± 10.3	p = 0.37
Weight (kg)	75.7 ± 13.6	77.5 ± 13.9	p = 0.23
BMI (kg/m^2^)	23.3 ± 2.11	23.4 ± 2.5	p = 0.11
Training hours (h/week)	18.5 ± 6.6	18.9 ± 6.3	p = 0.27
Training experience (year)	9.3 ± 3.8	8.8 ± 3.9	p = 0.28
*CAIT score*			
Left ankle	16.4 ± 5.5	24.8 ± 2.8	p < 0.001*
Right ankle	16.7 ± 5.8	25.1 ± 3.3	p < 0.001*
CAI ankle	15.4 ± 4.8 (513^#^)	–	–
Non-CAI ankle	23.8 ± 5.4 (81^#^)	25.5 ± 3.6	(96^#^)

*CAI* chronic ankle instability, *M* mean, *SD* standard deviation, *BMI* body mass index

^*^A significant difference between groups. ^#^Meaning the number of ankles

Gender influenced the presence of CAI (Table 2). Women had a higher prevalence of CAI than men (X(1)^2^ = 0.151, p = 0.003). When data was separated into two levels women had a higher prevalence of CAI than men at the college level (X(1)^2^ = 0.203, p = 0.001). No difference based on gender has been found at the semi-professional level (X(1)^2^ = 0.203, p = 0.47) (Table 2).

**Table 2:** The prevalence of chronic ankle instability between genders (n, %)

	Men (n = 243)		Women (n = 145)		Differences between gender
	CAI	Without CAI	CAI	Without CAI	
All participants (N = 388)	174 (72%)	69 (28%)	123 (85%)	22 (15%)	X^2^(1) = 0.151, p = 0.003*
College (n = 255)	99 (67%)	48 (32%)	92 (85%)	16 (15%)	X^2^(1) = 0.203, p = 0.001*
Semi-professional (n = 133)	75(78%)	21 (22%)	31 (84%)	6 (16%)	X^2^(1) = 0.203, p = 0.47

*CAI* chronic ankle instability

^*^p < 0.05

The competitive level did not influence the presence of CAI (*X*^*2*^ = 0.054, p = 0.29) (Table 3). When the data was separated into men and women, there was no difference of prevalence between different competitive levels among both genders (men: X^2^(1) = 0.117, p = 0.07, women: *X*^*2*^ = 0.017, p = 0.84).

**Table 3:** The prevalence of chronic ankle instability between different competitive levels (n, %)

	College (n = 255)		Semi-Professional (n = 133)		Differences between competitive level
	CAI	Without CAI	CAI	Without CAI	
All participants (N = 388)	191 (75%)	64 (25%)	106 (80%)	27 (20%)	X^2^(1) = 0.054, p = 0.29
men (n = 243)	99 (67%)	48 (33%)	75 (78%)	21 (22%)	X^2^(1) = 0.117, p = 0.07
women (n = 145)	92 (85%)	16 (15%)	31 (84%)	6 (16%)	X^2^(1) = 0.017, p = 0.84

*CAI* chronic ankle instability

For the three positions, the prevalence of CAI consisted of 76% for guard (124/164), 80% for forward (118/148), and 74% for center (51/69). The prevalence of CAI did not differ among positions (x^2^(2) = 0.55, p = 0.56) (Table 4).

**Table 4:** The prevalence of chronic ankle instability in different playing positions (n, %)

	Guard (n = 164)		Forward (n=148)		Center (n = 69)		
	CAI	Without CAI	CAI	Without CAI	CAI	Without CAI	
All (N = 381)	124 (76%)	40 (24%)	118 (80%)	30 (20%)	51 (74%)	18 (26%)	x^2^(2) = 0.55, p = 0.56
College (n = 248)	81 (74%)	29 (26%)	69 (77%)	21 (23%)	37 (77%)	11 (23%)	x^2^(2) = 0.04, p = 0.85
Semi-professional (n = 266)	43 (80%)	97 (20%)	49 (85%)	9 (15%)	14 (67%)	7 (33%)	x^2^(2) = 0.15, p = 0.22
Men (n = 238)	71 (70%)	31 (30%)	80 (78%)	23 (22%)	21 (64%)	12 (36%)	x^2^(2) = 0.21, p = 0.11
Women (n = 143)	53 (86%)	115 (16%)	38 (84%)	7 (16%)	30 (83%)	6 (17%)	x^2^(2) = 0.02, p = 0.96

*CAI* chronic ankle instability

## Discussion

The purpose of this study was (1) to investigate the prevalence of CAI in basketball athletes and (2) to assess if genders, competitive levels or positions influence this prevalence. The prevalence of CAI was high in the studied cohort. Gender affected the prevalence of CAI. Women showed a higher presence of CAI than men. The competitive level and position showed no influence on the prevalence of CAI.

### The prevalence of CAI

Regarding the first objective, the prevalence of CAI in this study cohort was high and above the prevalence of previous studies [23–25, 38]. Female basketball athletes in the current study presented an 85% (123/145) prevalence of CAI. Yet the previous studies showed 4% (2/26) among Japanese collegiate basketball athletes and 64% among female Australian netball athletes (61/96) [24, 38]. The movement patterns of netball are similar to basketball [38]. Collegiate basketball athletes in the current study displayed a 75% (191/255) prevalence of CAI but previous investigations showed 30% (17/57) among the US High school and collegiate basketball athletes; and 64% (14/22) and 36% (8/22) in Japanese collegiate basketball athletes [23, 25]. Different exclusion criteria applied in the current and previous studies cause discrepancies in results. The previous studies applied the exclusion criteria suggested by the International Ankle Consortium [3]. These criteria exclude participants with a history of previous surgeries, fractures or acute injuries in the lower extremities [25]. However, the current study included the participants who met the exclusion criteria.

There is a limitation when surveying the prevalence of CAI using the exclusion criteria defined by the International Ankle Consortium. If the participants with CAI are excluded because they have the issues mentioned above, the prevalence of CAI could be underestimated [23–25, 38]. On the other hand, if the participants without CAI have any conditions mentioned in the exclusion criteria, they may also perceive ankle instability. The perceived ankle instability might be owed to the other conditions instead of CAI. In this case, the prevalence of CAI would be overestimated. Therefore, the prevalence in the current study might be overestimated.

The rate of prevalence is almost twice as high when applying the exclusion criteria when investigating the prevalence of CAI [23]. Koshino et al. found a prevalence of 36% when excluding the participants who had other (confounding) conditions but the prevalence was 64% when they were not excluded [23]. Therefore, the real prevalence of CAI in Koshino’s and colleagues’ study might be in a range of 36% to 64%. In the current study the prevalence of CAI was 77%. It can be estimated that the prevalence might be 39% if participants were excluded based on the exclusion criteria from the International Ankle Consortium. Therefore, the true prevalence of CAI in the current study might be located between 39 and 77%. A standard method to identify the origin of perceived ankle instability is required to investigate the epidemiology of CAI; then participants with other conditions can be clearly categorized.

The high prevalence in the current study might be caused by the factors: preexisting perceived ankle instability, the period of the investigation and recovery conditions after ankle sprains. Preexisting perceived ankle instability might be a confounder when investigating the prevalence of CAI. In the current study, 30% of the participants without a history of ankle sprain showed perceived ankle instability (left ankle: 14/53 and right ankle 21/69). Therefore, there is a possibility that athletes with preexisting perceived ankle instability and a history of ankle sprain were categorized as CAI even though they regained the baseline level of perceived ankle stability after a severe ankle sprain. The current cross-sectional study cannot clarify if the perceived ankle instability is preexisting or caused by an ankle sprain.

The investigating period might be another explanation for the high prevalence of CAI. The current study was conducted one month before the start of the season. Most ankle injuries occur during the pre-season among collegiate basketball athletes [18]. In the pre-season, the training load intensity and duration are often higher than the in-season phase [39], leading to an increased risk of injury [40]. Recurrent ankle sprain is one of the signs of CAI. Therefore, a high rate of ankle injury in the pre-season may contribute to a high prevalence of CAI. In addition, data to depict the prevalence of CAI in different seasons is scarce. The optimal method to investigate overuse injury or chronic pain (e.g. the symptoms of low back pain, patellar tendinopathy or shoulder pain) is to perform a prospective longitudinal study and to measure the symptoms at regular intervals, since the chronic pain may fluctuate among different training seasons [41]. In the case of CAI, the CAI-related symptoms have not been measured continuously in athletic populations. Therefore, it is not clear if the presence of perceived ankle instability, frequency of recurrent ankle sprain or giving way fluctuates with time or training seasons as the other overuse injuries.

The inadequate recovery or lacking rehabilitation after an ankle sprain might also contribute to the high prevalence of CAI. Doherty et al. showed that after an acute ankle sprain, 40% of the participants who did not seek rehabilitation developed CAI, and 60% who did became ankle sprain copers [42]. Nevertheless, the effect of seeking rehabilitation showed no statistical significance on ankle status [42]. In addition, only half of the athletes sought a healthcare provider after an ankle sprain [25, 43]. Koshino et al. found that there are no ankle sprain copers in the basketball population and only 4.3–5.3% in the whole surveyed cohort [23]. Absence from practices and competitions due to injuries sustained during pre-season may affect participation in the in-season. Athletes might not recover properly after an ankle sprain and keep practicing with CAI. Secondary injuries could be developed with not properly healed tissue, such as sensori-perceptual or motor-behavioral impairments [7]. CAI affects the motor function of neuromuscular control and biomechanics on lower extremities, which might raise the risk of recurrent ankle sprains, cause post-traumatic osteoarthritis, increase the loading on the anterior cruciate ligament and further development of injuries [7]. Hence, managing CAI in the athletic population to prevent further unwanted injuries are essential.

### The prevalence of CAI and gender difference

The current study found that gender influences the prevalence of CAI. Female athletes had a higher prevalence of CAI than men at the collegiate level. This is consistent with previous work [25]. In contrast, a survey of a military cohort showed that men had a 2.33 times greater incidence of mild CAI than women [44]. The gender difference in the incidence of ankle sprains is also inconsistent. Two studies showed no gender difference between athletes in high schools and between the WNBA and NBA [15, 21]. One study found that male athletes in high school had a higher incidence of ankle injury than females [18].

The factors causing the differences in ankle injury rates between genders could be different anatomical structural, joint laxity and menstrual cycles [28, 29, 45–47]. Regarding anatomical structure, female collegiate athletes with an increased tibial varum and calcaneal eversion range of motion showed a greater risk of ankle sprain [48]. In the respect of joint laxity, women had a greater ligamentous laxity of the lateral ankle than men [28]. Regarding hormone fluctuation, women in the ovulating phase of menstrual cycles showed less postural stability than in the follicular phase [29, 46, 47]. Longitudinal studies are suggested to assess (1) the relation between a higher prevalence of CAI in women, (2) the underlying mechanism and (3) a specified prevention program for this population [41].

### The prevalence of CAI and different competitive levels

The current study found that the athlete’s competitive level did not relate to the prevalence of CAI. The relation between competitive level and ankle injury rate is not clear [20]. Two previous studies showed that athletes competing at a higher competitive level showed a lower prevalence of CAI [25, 38]. This may relate to more advanced skills and injury prevention measures applied to higher-level athletes [20, 49]. However, another study found that the rate of ankle sprains is higher among higher-level athletes than lower level [19]. Athletes at higher competitive levels may create more force when playing and this could result in a higher injury rate [30, 50].

The potential explanation for the inconsistent findings might be that athletes attend highly specialized training from a young age in Taiwan which is a risk factor for serious overuse injury [51]. Access to healthcare for young athletes was not common 10 years ago in Taiwan, which may lead to the development of CAI. Today 60% of the top ten high school basketball teams employ athletic trainers who provide injury care. The previously existing overuse injuries may last until they are at a higher level and cause the current result: a high prevalence of CAI.

### The prevalence of CAI and the different basketball positions

The current study found that the different positions showed no difference in the prevalence of CAI. To the best of the authors’ knowledge, no study has investigated the prevalence of CAI in different positions within basketball athletes. Regarding the relation of ankle injuries and positions, the outcome is inconsistent with previous works [18, 32, 34]. Previous studies found that athletes in the center and guard positions sustain the most ankle injuries. [18, 32, 34]. The center commonly has a size advantage for this position and needs to jump frequently for rebounds causing contact with other players and are therefore prone to suffer more injuries [34]. A guard in basketball sustains high physiological stress due to repetitive direction changes and may cause neuromuscular fatigue increasing the injury rate [18]. However, the current study and a previous study showed no difference between the different positions [32]. This might be due to the players' role being shared (e.g. guard position filling in for the forward position) and basic basketball movements. Although there are different basketball positions, the role of each athlete sometimes is not that distinct in Taiwan. Some athletes may play center and power forward at the same time and some play small forward and shooting guard at the same time. This may make the characteristics of the injury less distinguished. Besides, athletes in all positions perform the basic basketball movements including jumping, cuttings, rapid stop and sudden directional changes, which make an athlete prone to ankle sprains.

### Study limitations

There were some limitations to the study. Firstly, the current study is a cross-sectional design that portrays the prevalence of CAI among Taiwanese basketball athletes in pre-season. The prevalence is not representative for the whole season. Factors that affect the prevalence of CAI cannot be determined from the current data. In addition, the mechanical instability has not been examined so the participants with solely ankle pathological laxity may not be shown in the results. Finally, due to limited study resources no physician was present to differentiate between CAI and ankle instability caused by other issues. This may lead to an overestimation of the prevalence of CAI in the current study.

## Conclusion

An acute ankle sprain can cause the development of CAI, which can impact athletes negatively. In the current study, elite Taiwanese basketball athletes showed a higher prevalence of CAI than in previous studies. Female collegiate athletes have a higher prevalence of CAI than men. Competitive level and positions showed no difference in the prevalence of CAI. Prevention of chronic ankle instability should be a focus in basketball. Gender should be taken into consideration when applying the CAI prevention measures.

The recommendations for further studies investigating the prevalence of chronic ankle instability are: (1) prospective longitudinal study is recommended so the fluctuation of prevalence can be clearly depicted and the preexist perceived ankle instability will not confound the prevalence, (2) standard criteria for survey the prevalence of CAI should be developed, that can increase the precision of the prevalence, and (3) implementation of rehabilitation followed by ankle sprains should be reported.

## Supplementary Information

**Additional file 1.** Demographic characteristics of participants.

**Additional file 2.** Demographical differences between genders in collegiate athletes (n=255).

**Additional file 3.** Demographical differences between genders in the groups with and without chronic ankle instability (N=388).

**Additional file 4.** Demographical differences between genders in semi-professional athletes (n=133).

## Data Availability

The datasets used and/or analyzed during the current study are available from the corresponding author on reasonable request.

## Abbreviations

CAI: Chronic ankle instability
CAIT: The Cumberland Ankle Instability Tool
CAIT-TW: The Taiwan-Chinese version of the Cumberland Ankle Instability Tool
M: Mean
SD: Standard deviation
p: P-value
