# Supplementary material for: The prevalence of chronic ankle instability in basketball athletes: a cross-sectional study
Source: BMC Sports Sci Med Rehabil. 2022 Feb 18;14:27. doi: 10.1186/s13102-022-00418-0 (PMC8857785; doi:10.1186/s13102-022-00418-0)
Supplement: Supplementary file 1 — Additional file 1. Demographic characteristics of participants. [file 13102_2022_418_MOESM1_ESM.docx]

**Additional file 1 Demographic characteristics of participants**

|  | | All  (N=388) | | | | College  (n=255) | | | | Semi-Professional  (n=133) | | | | Men  (n=243) | | | | Women  (n=145) | | | | |
| --- | --- | --- | --- | --- | --- | --- | --- | --- | --- | --- | --- | --- | --- | --- | --- | --- | --- | --- | --- | --- | --- | --- |
|  | | M | ± | SD | Range | M | ± | SD | Range | M | ± | SD | Range | M | ± | SD | Range | M | ± | SD | Range |  |
| Age [year] | | 22.3 | ± | 3.8 | 18-37 | 20.1 | ± | 1.5 | 18-26 | 26.5 | ± | 3.4 | 19-37 | 22.5 | ± | 3.8 | 18-37 | 21.9 | ± | 3.9 | 18-37 |  |
| Height [cm] | | 179.9 | ± | 10.9 | 155-205 | 177.6 | ± | 10.8 | 155-205 | 184.4 | ± | 9.8 | 161-202 | 186 | ± | 7.5 | 163-205 | 169.8 | ± | 7.6 | 155-203 |  |
| Weight [kg] | | 76.1 | ± | 13.7 | 48-132 | 72.9 | ± | 13.2 | 48-132 | 82.3 | ± | 12.4 | 54-118 | 82.9 | ± | 11.1 | 19-34 | 64.7 | ± | 9.4 | 48-99 |  |
| BMI [kg/m^2^] | | 23.3 | ± | 2.2 | 18-34 | 22.9 | ± | 2.2 | 18-34 | 24.1 | ± | 2.0 | 20-30 | 23.9 | ± | 2.0 | 3-40 | 22.4 | ± | 2.1 | 18-32 |  |
| Training hours per week [hour] | | 18.6 | ± | 6.5 | 3-40 | 16.3 | ± | 5.4 | 3-40 | 23 | ± | 6.3 | 80-40 | 19.7 | ± | 6.2 | 0.5-21 | 16.8 | ± | 6.7 | 3-40 |  |
| Training experience [year] | | 9.2 | ± | 3.8 | 0.5-25 | 7.7 | ± | 2.9 | 0.5-14 | 12.1 | ± | 3.6 | 3-25 | 8.9 | ± | 3.7 | 3-30 | 9.8 | ± | 3.9 | 1-25 |  |
| Left CAIT-TW score | | 18.3 | ± | 6.1 | 1-30 | 18.8 | ± | 6.2 | 1-30 | 17.5 | ± | 6.1 | 4-30 | 19.3 | ± | 5.8 | 2-30 | 16.8 | ± | 6.4 | 1-30 |  |
| Right CAIT-TW score | | 18.7 | ± | 6.4 | 1-30 | 19.3 | ± | 6.5 | 1-30 | 17.5 | ± | 6.1 | 2-30 | 19.5 | ± | 6.0 | 1 | 17.3 | ± | 6.8 | 1-30 |  |
| Unilateral CAI [n (%)] | | 102 (26) | | | | 75 (29) | | | | 27 (20) | | | | 62 (26) | | | | 40 (28) | | | | |
| Bilateral CAI [n (%)] | | 195 (50) | | | | 116 (46) | | | | 79 (59) | | | | 112 (46) | | | | 83 (57) | | | | |
| without CAI [n (%)] | | 91 (24) | | | | 64 (25) | | | | 27 (20) | | | | 69 (28) | | | | 22 (15) | | | | |
| Having history of significant ankle sprain(s) [n] | Left | 335 | | | | 213 | | | | 122 | | | | 207 | | | | 128 | | | | |
|  | Right | 319 | | | | 202 | | | | 117 | | | | 193 | | | | 126 | | | | |
| Experiencing giving way [n]  (≥2 in the past 6 months) | Left | 190 | | | | 135 | | | | 135 | | | | 121 | | | | 69 | | | | |
|  | Right | 181 | | | | 128 | | | | 128 | | | | 111 | | | | 70 | | | | |
| Experiencing recurrent ankle sprain [n] | Left | 251 | | | | 167 | | | | 97 | | | | 160 | | | | 104 | | | | |
|  | Right | 251 | | | | 150 | | | | 101 | | | | 151 | | | | 100 | | | | |

*M: mean, SD: standard deviation, CAIT-TW: score of the Taiwan-Chinese version of the Cumberland Ankle Instability Tool. CAI: chronic ankle instability*
