# Supplementary material for: The prevalence of chronic ankle instability in basketball athletes: a cross-sectional study
Source: BMC Sports Sci Med Rehabil. 2022 Feb 18;14:27. doi: 10.1186/s13102-022-00418-0 (PMC8857785; doi:10.1186/s13102-022-00418-0)
Supplement: Supplementary file 2 — Additional file 2. Demographical differences between genders in collegiate athletes (n=255). [file 13102_2022_418_MOESM2_ESM.docx]

Additional file 2 Demographical differences between genders in collegiate athletes (n=255)

|  |  | CAI (n=191) | | | | | | | | | without CAI (n=64) | | | | | | | | | |
| --- | --- | --- | --- | --- | --- | --- | --- | --- | --- | --- | --- | --- | --- | --- | --- | --- | --- | --- | --- | --- |
|  |  | Men (n=99) | | | | Women (n=92) | | | |  | Men (n=48) | | | | Women (n=16) | | | |  | |
|  |  | M | ± | SD | range | M | ± | SD | range | genders difference | M | ± | SD | range | M | ± | SD | range | genders difference |  |
| Age [year] | | 20.1 | ± | 1.4 | 18-24 | 20.3 | ± | 1.7 | 18-24 | 0.36 | 20.1 | ± | 1.4 | 18-22 | 19.6 | ± | 1.3 | 18-22 | 0.11 |  |
| Height [cm] | | 184.7 | ± | 7.3 | 163-205 | 168.4 | ± | 7.6 | 155-203 | 0.79 | 183.7 | ± | 7.4 | 170-200 | 168.8 | ± | 5.9 | 158-178 | 0.04* |  |
| Weight [kg] | | 80.5 | ± | 10.8 | 60-115 | 62.8 | ± | 8.1 | 48-99 | <0.001* | 79.5 | ± | 11.9 | 60-132 | 63.4 | ± | 9.7 | 48-91 | <0.001* |  |
| BMI [kg/m^2^] | | 23.5 | ± | 2.0 | 19-31 | 22.1 | ± | 1.9 | 18-32 | <0.001* | 23.5 | ± | 2.4 | 20-34 | 22.2 | ± | 2.7 | 19-29 | =0.001* |  |
| Training hours [hour/week] | | 17.2 | ± | 4.6 | 10-28 | 15 | ± | 6 | 3-40 | =0.001* | 16.6 | ± | 5.7 | 3-35 | 17.2 | ± | 4.2 | 12-30 | 0.90 |  |
| Training experience [year] | | 7.0 | ± | 2.5 | 0.5-12 | 8.4 | ± | 3.2 | 1-14 | <0.001* | 7.1 | ± | 3 | 1-14 | 8.8 | ± | 2.7 | 4-13 | 0.43 |  |
| CAIT score | Left | 17.9 | ± | 5.1 | 3-30 | 15.5 | ± | 5.7 | 1-30 | 0.003* | 24.8 | ± | 3.3 | 16-30 | 25.4 | ± | 2.4 | 22-30 | 0.73 |  |
|  | Right | 18.0 | ± | 5.0 | 3-30 | 16.3 | ± | 6.5 | 1-30 | 0.10 | 25.3 | ± | 3.8 | 16-30 | 26.2 | ± | 3.1 | 22-30 | 0.77 |  |
|  | CAI | 16.6 |  | 3.8 | (161) ^#^ | 14.8 |  | 5.3 | (162) ^#^ | -- |  | - |  | - |  | - |  |  | - |  |
|  | without CAI | 23.9 |  | 5.4 | (37) ^#^ | 23.9 |  | 6.1 | (22) ^#^ | -- | 24.9 |  | 3.1 | (138) | 25.1 |  | 2.9 | (44) ^#^ | - |  |

CAI: chronic ankle instability, M: mean, SD: standard deviation, BMI: body mass index, CAIT score: score of the Cumberland Ankle Instability Tool, *: showing a significant difference between genders. ^#^: meaning the number of ankles.
