# Supplementary material for: The prevalence of chronic ankle instability in basketball athletes: a cross-sectional study
Source: BMC Sports Sci Med Rehabil. 2022 Feb 18;14:27. doi: 10.1186/s13102-022-00418-0 (PMC8857785; doi:10.1186/s13102-022-00418-0)
Supplement: Supplementary file 3 — Additional file 3. Demographical differences between genders in the groups with and without chronic ankle instability (N=388). [file 13102_2022_418_MOESM3_ESM.docx]

Additional file 3 Demographical differences between genders in the groups with and without chronic ankle instability (N=388)

|  |  | CAI (n=297) | | | | | | | | | without CAI (n=118) | | | | | | | | | |
| --- | --- | --- | --- | --- | --- | --- | --- | --- | --- | --- | --- | --- | --- | --- | --- | --- | --- | --- | --- | --- |
|  |  | Men (n=174) | | | | Women (n=123) | | | |  | Men (n=69) | | | | Women (n=22) | | | |  | |
|  |  | M | ± | SD | range | M | ± | SD | range | genders difference | M | ± | SD | range | M | ± | SD | range | genders difference |  |
| Age [year] | | 22.6 | ± | 3.7 | 18-33 | 22.1 | ± | 4.0 | 18-37 | 0.10 | 22.3 | ± | 4.0 | 18-37 | 20.8 | ± | 2.8 | 18-29 | 0.11 |  |
| Height [cm] | | 186.2 | ± | 7.3 | 163-205- | 169.9 | ± | 7.9 | 155-203 | <0.001* | 185.5 | ± | 8.0 | 170-202 | 169.1 | ± | 5.6 | 158-178 | 0.04* |  |
| Weight [kg] | | 83.3 | ± | 10.6 | 60-118 | 64.9 | ± | 9.5 | 48-99 | <0.001* | 82.0 | ± | 12.2 | 60-132 | 63.5 | ± | 8.6 | 48-91 | <0.001* |  |
| BMI [kg/m^2^] | | 23.9 | ± | 1.9 | 19-31 | 22.4 | ± | 2.1 | 18-32 | <0.001* | 23.7 | ± | 2.4 | 20-34 | 22.2 | ± | 2.4 | 19-29 | 0.001* |  |
| Training hours [hour/week] | | 20.0 | ± | 6.1 | 10-40 | 16.4 | ± | 6.8 | 3-40 | <0.001* | 18.9 | ± | 6.5 | 3-35 | 18.9 | ± | 5.8 | 12-30 | 0.90 |  |
| Training experience [year] | | 9.0 | ± | 3.6 | 0.5-20 | 9.8 | ± | 4 | 1-25 | 0.049* | 8.6 | ± | 4.1 | 1-21 | 9.5 | ± | 3.4 | 4-19 | 0.21 |  |
| CAIT score | Left | 17.1 | ± | 5.2 | 3-30 | 15.3 | ± | 5.8 | 1-30 | 0.01* | 24.7 | ± | 2.9 | 16-30 | 24.9 | ± | 2.3 | 22-30 | 0.73 |  |
|  | Right | 17.3 | ± | 5.4 | 2-30 | 15.9 | ± | 6.3 | 1-30 | 0.04* | 25.0 | ± | 3.3 | 16-30 | 25.3 | ± | 3.4 | 18-30 | 0.77 |  |
|  | CAI | 16.0 | ± | 4.4 | (296)^#^ | 14.6 | ± | 5.2 | (217) ^#^ | - |  | - |  |  |  | - |  | - | - |  |
|  | without CAI | 24.2 | ± | 5.0 | (52) ^#^ | 23.3 | ± | 6.4 | (29) ^#^ | - | 24.9 | ± | 3.1 | (138) | 25.1 | ± | 2.9 | (44) ^#^ | - |  |

CAI: chronic ankle instability, M: mean, SD: standard deviation, BMI: body mass index, CAIT score: score of the Cumberland Ankle Instability Tool, *: showing a significant difference between genders. #: meaning the number of ankles.
