# Supplementary material for: The prevalence of chronic ankle instability in basketball athletes: a cross-sectional study
Source: BMC Sports Sci Med Rehabil. 2022 Feb 18;14:27. doi: 10.1186/s13102-022-00418-0 (PMC8857785; doi:10.1186/s13102-022-00418-0)
Supplement: Supplementary file 4 — Additional file 4. Demographical differences between genders in semi-professional athletes (n=133). [file 13102_2022_418_MOESM4_ESM.docx]

Additional file 4 Demographical differences between genders in semi-professional athletes (n=133)

|  |  | CAI (n=106) | | | | | | | | | without CAI (n=27) | | | | | | | | | |
| --- | --- | --- | --- | --- | --- | --- | --- | --- | --- | --- | --- | --- | --- | --- | --- | --- | --- | --- | --- | --- |
|  |  | Men (n=75) | | | | Women (n=31) | | | |  | Men (n=21) | | | | Women (n=6) | | | |  | |
|  |  | M | ± | SD | range | M | ± | SD | range | genders difference | M | ± | SD | range | M | ± | SD | range | genders difference |  |
| Age [year] | | 26.0 | ± | 2.9 | 20-33 | 27.4 | ± | 4.2 | 20-37 | 0.19 | 27.4 | ± | 3.5 | 23-37 | 23.8 | ± | 3.5 | 19-29 | 0.06 |  |
| Height [cm] | | 188.2 | ± | 6.9 | 170-200 | 174.2 | ± | 7.1 | 161-186 | <0.001* | 189.6 | ± | 8.1 | 174-202 | 170.0 | ± | 4.9 | 164-178 | 0.14 |  |
| Weight [kg] | | 87.0 | ± | 9.1 | 70-118 | 71.1 | ± | 10.7 | 54-93 | <0.001* | 87.6 | ± | 11.2 | 68-118 | 63.8 | ± | 5.6 | 60-75 | 0.12 |  |
| BMI [kg/m^2^] | | 24.5 | ± | 1.6 | 22-30 | 23.3 | ± | 2.4 | 20-29 | 0.01* | 24.3 | ± | 2.3 | 21-30 | 22.1 | ± | 1.0 | 21-24 | 0.16 |  |
| Training hours [hour/week] | | 23.7 | ± | 5.9 | 10-40 | 20.5 | ± | 7.3 | 8-30 | 0.02* | 24.0 | ± | 5.1 | 10-30 | 23.3 | ± | 7.5 | 15-30 | 0.89 |  |
| Training experience [year] | | 11.6 | ± | 3.1 | 3-20 | 13.9 | ± | 3.5 | 9-25 | 0.003* | 11.7 | ± | 4.4 | 5-21 | 11.3 | ± | 4.5 | 7-19 | 0.86 |  |
| CAIT score | Left | 16.0 | ± | 5.3 | 4-30 | 15.0 | ± | 5.9 | 6-28 | 0.27 | 24.6 | ± | 1.9 | 22-30 | 23.7 | ± | 1.6 | 22-26 | 0.35 |  |
|  | Right | 16.3 | ± | 5.7 | 2-30 | 14.5 | ± | 5.4 | 4-29 | 0.09 | 24.4 | ± | 1.5 | 22-28 | 22.8 | ± | 3.3 | 18-27 | 0.03* |  |
|  | CAI | 15.2 |  | 4.8 | (135)^#^ | 13.9 |  | 4.8 | (55)^#^ | - |  | - |  |  |  | - |  | - | - |  |
|  | without CAI | 24.2 |  | 5.0 | (15)^#^ | 23.3 |  | 6.4 | (7)^#^ | - | 24.8 |  | 1.9 | (20)^#^ | 23.2 |  | 2.5 | (12)^#^ | - |  |

CAI: chronic ankle instability, M: mean, SD: standard deviation, BMI: body mass index, CAIT score: score of the Cumberland Ankle Instability Tool, *: showing a significant difference between genders. #: meaning the number of ankles.
